# Individualized antiresorptive therapy in fibrous dysplasia and McCune-Albright syndrome: A retrospective cohort study

**DOI:** 10.1016/j.bonr.2026.101933

**Published:** 2026-06-19

**Authors:** Tonio Lipkow, Mikolaj Bartosik, Johann Sprick-Schütte, Florian Barvencik, Michael Amling, Ralf Oheim

**Affiliations:** a Department of Osteology and Biomechanics, University Medical Center Hamburg-Eppendorf, Hamburg, Germany

**Keywords:** Bone metabolism, Denosumab, Fibrous dysplasia, McCune-Albright syndrome, Rebound, Zoledronate

## Abstract

Fibrous dysplasia/McCune-Albright syndrome (FD/MAS) is a rare benign bone disorder caused by postzygotic mutations in *GNAS*, characterized by skeletal lesions leading to pain, deformities, and fractures. Although antiresorptive agents such as denosumab and zoledronate are used, evidence remains limited, and denosumab carries a well-known rebound risk. Given the heterogeneous disease activity in FD/MAS, standardized treatment approaches are frequently insufficient, raising the need for individualized treatment strategies. We therefore evaluated the effects and safety of individualized denosumab-zoledronate therapy with clinically guided treatment adjustments. A single-center FD/MAS cohort (*n* = 42) managed between 2014 and 2025 was retrospectively analyzed. Clinical, radiographic, histopathological, and baseline biochemical characteristics were assessed in the overall cohort, whereas longitudinal analyses of treatment response, laboratory markers, pain scores, and adverse events were restricted to an antiresorptive-treated subgroup of 11 patients. Compared with untreated patients, the treated subgroup showed higher skeletal lesion burden and elevated osteocalcin levels. During therapy, elevated bone turnover markers decreased, particularly ALP (−44.3%, *p* = 0.016). Most treated patients showed stable biochemical trajectories and reported pain reduction under therapy. One young MAS patient experienced severe rebound hypercalcemia (4.43 mmol/L) with renal failure after delayed denosumab administration. In conclusion, individualized antiresorptive therapy with denosumab and zoledronate was associated with symptomatic improvement and largely stable clinical courses in most treated FD/MAS patients. However, denosumab treatment may carry a clinically relevant rebound risk, particularly in young MAS patients with extensive skeletal burden and high bone turnover. Further studies are needed to establish specific, safe and effective treatment strategies.

## Highlights

• Treatment patterns varied with individualized antiresorptive therapy in FD/MAS.
• Pain outcomes suggested potential symptomatic benefit of antiresorptive therapy.
• Most treated patients showed clinically manageable disease courses.
• Rebound hypercalcemia followed delayed denosumab in a severely affected MAS patient.

## 1 Introduction

Fibrous dysplasia (FD) is a rare, benign skeletal disorder in which physiological bone and bone marrow are replaced by fibro-osseous tissue, resulting in pain, bone deformities, and pathological fractures (Boyce and Collins, 2020). FD may occur in a monostotic (MFD) or polyostotic form (PFD). When FD is associated with endocrinopathies or characteristic hyperpigmented skin lesions (formerly described as café-au-lait), the condition is termed McCune-Albright syndrome (MAS), and in combination with intramuscular myxomas, it is referred to as Mazabraud syndrome (MS) (Boyce and Collins, 2020; Collins et al., 2012). FD/MAS is caused by postzygotic activating variants in the *GNAS* gene, which encodes the α-subunit of the stimulatory G protein (Gsα) (Boyce and Collins, 2020; Schwindinger et al., 1992; Weinstein et al., 1991). These variants impair the differentiation of osteoprogenitor cells, leading to dysfunctional osteoblasts and the formation of immature woven bone (Zhao et al., 2018; Marie et al., 1997). In parallel, increased secretion of cytokines such as interleukin-6 (IL-6) and receptor activator of nuclear factor kappa B ligand (RANKL) stimulates osteoclast activity, resulting in excessive bone resorption (Yamamoto et al., 1996; de Castro et al., 2019). The disrupted homeostasis of bone formation and resorption ultimately leads to characteristic FD lesions with immature trabeculae embedded in a fibrous stroma (Boyce and Collins, 2020; Riminucci et al., 1997).

Current treatment options are primarily symptomatic, consisting of surgical interventions, analgesic management, and the use of antiresorptive agents (Javaid et al., 2019; Bertin et al., 2023; Trojani et al., 2023). Among these antiresorptive agents, denosumab acts as a fully human monoclonal antibody against RANKL, whereas zoledronate is a third-generation intravenous bisphosphonate that inhibits osteoclast activity and survival. In small case series and retrospective cohort studies, both agents have shown promising results in FD/MAS, including reductions in pain and biochemical markers of bone turnover (Trojani et al., 2023; Majoor et al., 2019a; Valadares et al., 2022; Tripathy et al., 2020). However, evidence remains limited, treatment regimens are highly heterogeneous, and data on sequential or combined use of both agents remain scarce. In addition, discontinuation of denosumab can trigger a rebound phenomenon, a well-documented problem in osteoporosis treatment, which in patients with FD/MAS may result in severe hypercalcemia (Walker and Shane, 2023; Meier et al., 2021) due to a subsequent increase in bone resorption. In contrast, zoledronate has not been linked to rebound risk and is even applied in osteoporosis therapy to prevent rebound following denosumab withdrawal (Everts-Graber et al., 2020; Grassi et al., 2024).

Given the marked heterogeneity of FD/MAS regarding skeletal burden, pain, and biochemical activity, individualized treatment decisions are frequently required in clinical practice. In some patients, a single antiresorptive agent may be insufficient to adequately control symptoms, biochemical activity, or rebound phenomena. However, current evidence does not adequately reflect these longitudinal real-world treatment strategies, particularly regarding individualized antiresorptive therapy and rebound management in FD/MAS.

Therefore, the aim of the present study was to characterize the clinical and biochemical effects and safety of individualized antiresorptive therapy, including denosumab and zoledronate, in a German single-center cohort of patients with FD/MAS, with particular focus on rebound phenomena.

## 2 Methods

### 2.1 Study design

We conducted a retrospective cohort study of 42 patients with FD who were managed at our specialized outpatient clinic between 2014 and 2025. All consecutive patients with confirmed FD/MAS presenting during the study period were included. Diagnoses of FD and MAS, or MS, were based on clinical and radiographical findings, supplemented by histological and genetic analyses when indicated. The subdivision into MFD, PFD, MAS and MS is presented in Table 1. The patients underwent baseline laboratory assessments, and as part of routine clinical workup, in some patients, additional investigations beyond radiographic imaging were performed, including dual-energy X-ray absorptiometry (DXA) as well as high-resolution peripheral quantitative computed tomography (HR-pQCT). A subgroup of 11 patients received antiresorptive therapy with denosumab 60 mg subcutaneous (sc) and/or zoledronate 5 mg intravenous (iv) in individualized treatment approaches. Treatment initiation and choice of agent were based on disease burden, persistent bone pain, biochemical activity, and prior treatment response. Zoledronate was generally used as first-line antiresorptive therapy, whereas denosumab was considered in cases of insufficient symptomatic or biochemical response. Treatment adjustments, including switching or sequential therapy, were guided by clinical course, laboratory monitoring, and rebound risk. However, therapeutic decisions ultimately remained individualized because of the heterogeneous clinical presentation of FD/MAS. Analyses within the overall cohort focused on descriptive clinical, radiographic, histopathological, and biochemical characterization. In contrast, longitudinal analyses of treatment response, biochemical trajectories, pain scores, adverse events, and rebound phenomena were restricted to the antiresorptive-treated subgroup of 11 patients. This study was conducted in accordance with local ethical guidelines and the Declaration of Helsinki.

**Table 1:** Baseline cohort characteristics. Abbreviations: monostotic fibrous dysplasia (MFD), polyostotic fibrous dysplasia (PFD), McCune-Albright syndrome (MAS), Mazabraud syndrome (MS), body mass index (BMI), visual analogue scale (VAS), alkaline phosphatase (ALP), osteocalcin (OC), and urinary deoxypyridinoline/creatinine (DPD/Cr). FD localization: axial = bones of the trunk skeleton (spine, ribs, sternum, pelvis); appendicular = bones of the extremities including the shoulder girdle; craniofacial = skull and facial bones. Statistically significant differences are shown with exact p-values in bold.

Baseline cohort characteristics					
	MFD (n = 21)	PFD (n = 10)	MAS (n = 9)	MS (n = 2)	P-value^a^
Gender, N (%)					
Female	13 (61.9)	7 (70.0)	5 (55.6)	1 (50.0)	
Male	8 (38.1)	3 (30.0)	4 (44.4)	1 (50.0)	
Age (years), mean (SD)	44.24 (17.70)	42.40 (18.86)	26.56 (7.88)	39.50 (13.44)	**0.031**
Age at first diagnosis (years), mean (SD)	38.63 (17.17)	44.40 (24.75)	17.14 (16.09)	31.50 (0.71)	**0.026**
BMI (kg/m^2^), mean (SD)	25.48 (5.49)	26.94 (7.10)	24.10 (4.37)	21.50 (0.42)	0.574
FD localization, N (%)					
Axial	2 (9.5)	6 (60.0)	7 (77.8)	1 (50.0)	
Appendicular	9 (42.9)	4 (40.0)	8 (88.9)	2 (100.0)	
Craniofacial	9 (42.9)	6 (60.0)	3 (33.3)	2 (100.0)	
Prevalent fractures, mean (SD)	0.94 (1.00)	1.78 (1.92)	3.00 (3.32)	0.50 (0.71)	0.105
Pain, mean (SD)					
VAS (1–10)	4.63 (2.87)	7.00 (1.41)	4.89 (2.03)	9.00 (0)	0.136
Bone turnover markers, mean (SD)					
ALP (U/L)	75.25 (23.00)	124.40 (104.81)	180.6 (132.70)	87.00 (25.46)	**0.032**
OC (μg/L)	18.95 (5.29)	28.54 (29.59)	62.72 (34.76)	22.00 (2.40)	**<0.001**
DPD/Cr (nmol/mmol)	6.35 (2.28)	12.20 (16.31)	12.63 (10.53)	12.50 (2.12)	0.163
Specific therapy, N (%)					
Denosumab	1 (4.8)	2 (20.0)	4 (44.4)	1 (50.0)	
Zoledronate	1 (4.8)	1 (10.0)	5 (55.6)	1 (50.0)	

a Group comparisons were performed for MFD, PFD, and MAS; MS was excluded due to small sample size.

### 2.2 Bone metabolism

Biochemical analyses were conducted in the certified local laboratory (Institute for Clinical Chemistry and Laboratory Medicine, University Medical Center Hamburg-Eppendorf, Germany). During routine outpatient visits, parameters of calcium and phosphate homeostasis included plasma calcium, phosphate, and alkaline phosphatase (ALP), as well as serum intact fibroblast growth factor 23 (FGF23), 25-hydroxyvitamin D (25(OH)D), and parathyroid hormone (PTH). Bone formation markers comprised serum procollagen type 1 N-terminal propeptide (P1NP), bone-specific alkaline phosphatase (bALP), and osteocalcin (OC), whereas bone resorption was evaluated using serum collagen type I C-terminal telopeptide (CTX) and urinary deoxypyridinoline/creatinine (DPD/Cr).

### 2.3 Pain assessment

Pain was assessed using the visual analogue scale (VAS) during outpatient visits at baseline and follow-up, when feasible. Baseline VAS refers to the first documented pain assessment during the observation period. The VAS records the patient's current pain on a scale from 0 (no pain) to 10 (worst imaginable pain). Quantitative VAS data were available for 9 patients within the treated subgroup. In addition, longitudinal changes in pain during the course of treatment were qualitatively assessed based on patient-reported outcomes documented in clinical records and categorized as improved or not improved.

### 2.4 Statistical analysis

Statistical analysis was performed using JASP version 0.19.3 (JASP Team, University of Amsterdam, Netherlands) and GraphPad Prism version 10.3.1 (GraphPad Software, San Diego, CA, USA). Data distribution was evaluated using the Shapiro-Wilk test. Parametric tests were applied when normality was confirmed, otherwise non-parametric tests were chosen. For two-group comparisons, Student's *t*-test was used, or the Mann-Whitney *U* test in case of non-normally distributed data. Paired samples were analyzed using the paired *t*-test or the Wilcoxon signed-rank test. For comparisons involving more than two groups, parametric data were analyzed using analysis of variance (ANOVA) with Šídák-adjusted multiple comparisons, whereas non-parametric data were assessed using the Kruskal-Wallis test followed by Dunn's multiple comparisons. Linearity between variables was evaluated by visual inspection of scatterplots and verified using simple linear regression. Correlation analyses were performed using Pearson's correlation coefficient for normally distributed data, and Spearman's rank correlation for non-normally distributed data. Statistical significance was set at *p* < 0.05.

## 3 Results

### 3.1 Cohort characteristics

**Fig. 1:**
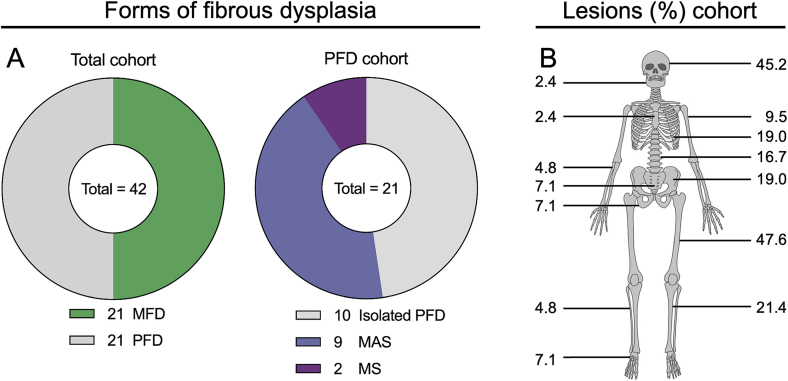
Cohort composition and skeletal lesion distribution in fibrous dysplasia (FD). (A) Distribution of monostotic (MFD) and polyostotic FD (PFD), McCune-Albright syndrome (MAS), and Mazabraud syndrome (MS). (B) Distribution of FD lesions across skeletal localizations, expressed as the percentage of patients affected at each site.

Baseline characteristics are summarized in Table 1. The cohort (*n* = 42) consisted of 50.0% (*n* = 21) MFD and 50.0% (n = 21) PFD (Fig. 1A). Skeletal involvement was most frequent in the appendicular skeleton (*n* = 23, 54.8%), while craniofacial lesions were present in nearly half of the cohort (*n* = 20, 47.6%) (Fig. 1B). Baseline bone turnover markers were highest in MAS patients, with significant group differences observed for ALP (*p* = 0.032) and OC (*p* < 0.001), but no significant differences in DPD/Cr (*p* = 0.163) (Table 1). During follow-up, 11 of 42 patients (26.2%) received antiresorptive treatment with denosumab and/or zoledronate, with MAS patients representing the largest proportion of treated individuals (*n* = 6, 54.5%) (Table 1). Compared with untreated patients, the treated subgroup showed a higher lesion burden (*p* = 0.008) and higher OC levels (*p* = 0.025). Additional data on bone turnover markers are available in Supplementary Table 1. Endocrine manifestations were generally co-managed by treating endocrinologists according to clinical need. No documented cases of growth hormone excess were identified during the observation period. Furthermore, representative radiological and histological findings of FD/MAS, including typical ground-glass lesions and characteristic fibro-osseous histology with irregular woven bone trabeculae embedded in fibrous stroma, are shown in Supplementary Fig. 1.

### 3.2 Associations between skeletal burden, pain and bone turnover

**Fig. 2:**
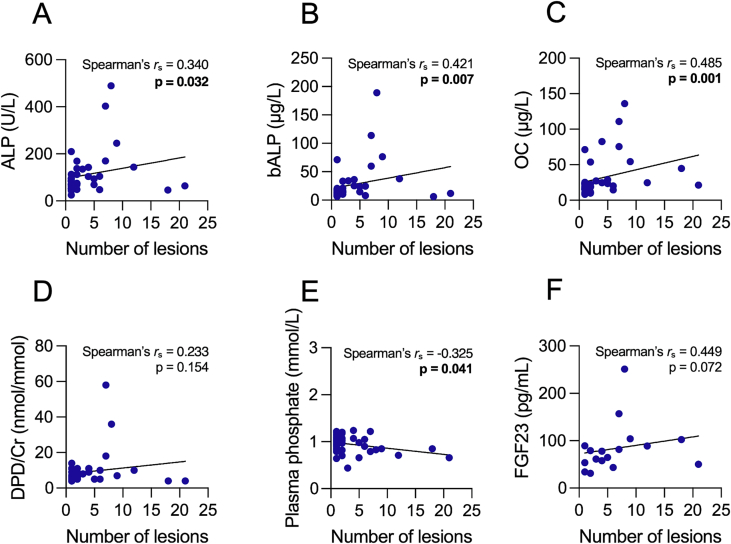
Associations between skeletal burden and baseline bone turnover. (A–D) Correlations between the number of fibrous dysplasia (FD) lesions and baseline bone turnover markers, including alkaline phosphatase (ALP), bone-specific alkaline phosphatase (bALP), osteocalcin (OC), and urinary deoxypyridinoline/creatinine (DPD/Cr). (E–F) Additional correlations with parameters of phosphate metabolism: plasma phosphate and intact fibroblast growth factor 23 (FGF23). Statistically significant correlations are shown with exact *p*-values in bold.

At baseline, both ALP and bALP, markers of bone formation, showed weak-to-moderate positive correlations with the number of lesions within the overall cohort (ALP: r_s_ = 0.340, *p* = 0.032; bALP: r_s_ = 0.421, *p* = 0.007) (Fig. 2A–B). Osteocalcin (OC) also showed a significant moderate positive correlation (r_s_ = 0.485, *p* = 0.001), whereas no significant association was observed for the bone resorption marker DPD/Cr (r_s_ = 0.233, *p* = 0.154) (Fig. 2C–D). In contrast, plasma phosphate was negatively associated with lesion number (rs = −0.325, *p* = 0.041) (Fig. 2E). FGF23 did not correlate significantly with lesion number (rs = 0.449, *p* = 0.072) (Fig. 2F). In addition, correlation analyses between pain scores and bone turnover parameters within the overall cohort revealed only an isolated moderate positive association with DPD levels (rs = 0.366, *p* = 0.043), whereas no consistent correlations were observed for other bone turnover markers or mineral metabolism parameters (Suppl. Fig. 2).

### 3.3 Biochemical and clinical response to antiresorptive treatment

The characteristics of the treated subgroup (*n* = 11) and the therapy schemes are summarized in Table 2. Five patients (45.5%) required individualized sequential therapy with denosumab and zoledronate due to insufficient control of bone turnover, calcium and phosphate homeostasis, or disease-related symptoms under monotherapy. The remaining patients responded to a single antiresorptive agent. Additionally, two of the treated patients had a concurrent diagnosis of osteoporosis (Table 2). In the treated subgroup, some patients had received antiresorptive therapy prior to initiation of denosumab/zoledronate treatment. However, no additional antiresorptive agents were administered during follow-up. Supportive treatments such as vitamin D_3_ supplementation, analgesics, pain-modulating medications, and physiotherapy were used in both groups according to clinical need. Overall, there were no cases of rare antiresorptive-related adverse events, such as osteonecrosis of the jaw, recurrent infections, symptomatic hypocalcemia, or atypical femoral fractures.

**Table 2:** Treated subgroup characteristics. Abbreviations: female (F), male (M), fibrous dysplasia (FD), monostotic fibrous dysplasia (MFD), polyostotic fibrous dysplasia (PFD), McCune-Albright syndrome (MAS), Mazabraud syndrome (MS), bisphosphonate (BP), denosumab (Dmab), zoledronate (Zol), subcutaneous (sc), intravenous (iv), alkaline phosphatase (ALP), urinary deoxypyridinoline/creatinine (DPD/Cr), visual analogue scale (VAS), and not available (NA). Patients marked with an asterisk (*) had a concurrent diagnosis of osteoporosis.

Treated subgroup characteristics									
Patient no.	Sex/age (years)	Type of FD	Previous BP therapy	Antiresorptive therapy (dose)	FD lesions (number)	Baseline ALP (U/L)	Baseline DPD/Cr (nmol/mmol)	Pain	
								Baseline VAS (0–10)	Improvement
1	M/19	MAS	Pamidronate	Dmab (60 mg; sc), Zol (5 mg; iv)	8	489	36	4	Yes
2	F/49	MS	Pamidronate	Dmab (60 mg; sc), Zol (5 mg; iv)	6	105	10	9	Yes
3*	F/60	MFD	/	Dmab (60 mg; sc)	2	NA	NA	NA	NA
4*	M/75	PFD	/	Dmab (60 mg; sc)	2	75	11	NA	NA
5	M/34	MAS	Risedronate	Dmab (60 mg; sc), Zol (5 mg; iv)	2	49	5	8	Yes
6	M/18	MAS	/	Dmab (60 mg; sc), Zol (5 mg; iv)	4	143	9	7	Yes
7	M/26	MAS	/	Zol (5 mg; iv)	9	245	10	6	No
8	F/43	PFD	Alendronate	Dmab (60 mg; sc), Zol (5 mg; iv)	12	144	10	7	Yes
9	F/26	MAS	Pamidronate	Zol (5 mg; iv)	2	138	NA	5	No
10	M/22	MFD	/	Zol (5 mg; iv)	1	97	4	2	Yes
11	F/39	MAS	Pamidronate	Dmab (60 mg; sc)	18	46	4	2	Yes

**Fig. 3:**
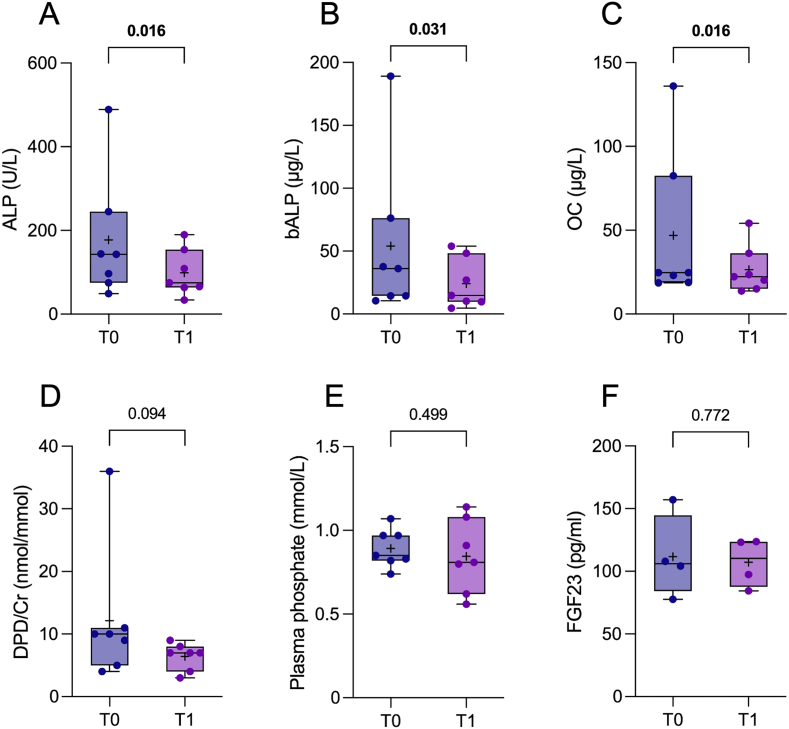
Effect of specific antiresorptive therapy on bone metabolism. (A-D) Changes in bone turnover markers before therapy (T0) and during therapy (T1; mean follow-up 16 months). Shown are alkaline phosphatase (ALP), bone-specific alkaline phosphatase (bALP), osteocalcin (OC), and urinary deoxypyridinoline/creatinine (DPD/Cr). (E-F) Additional parameters of phosphate metabolism: plasma phosphate and intact fibroblast growth factor 23 (FGF23). Laboratory values are displayed as median (line). Statistically significant differences are shown with exact p-values in bold.

During therapy, laboratory follow-up assessments were performed repeatedly according to clinical course and treatment response, generally at intervals of approximately three months after treatment initiation. For standardized longitudinal analyses, one representative follow-up measurement obtained during therapy at a comparable interval was selected for each evaluable patient, resulting in a mean observation period of 16 ± 2 months. Among patients with sufficiently comparable pre-treatment and on-treatment measurements, markers of bone formation decreased significantly (ALP: *p* = 0.016; bALP: *p* = 0.031; OC: p = 0.016), whereas the bone resorption marker DPD/Cr did not change significantly (*p* = 0.094) (Fig. 3A–D). Parameters of phosphate metabolism, including plasma phosphate and FGF23, also remained stable (plasma phosphate: *p* = 0.499; FGF23: *p* = 0.772) (Fig. 3E–F). Corresponding individual pre-therapy-to-follow-up trajectories are provided in Supplementary Fig. 3.

Clinically, the majority of patients experienced pain relief during the course of antiresorptive treatment. More specifically, 77.8% (*n* = 7 of 9) of treated patients reported decreased pain, while data were missing for two patients, suggesting a potential effect of antiresorptives beyond their impact on bone metabolism. The two patients without reported pain improvement were both MAS patients with chronic pain conditions treated with zoledronate monotherapy and one of whom had a documented comorbid psychological condition.

### 3.4 Individualized long-term therapy patterns and risks

Symptomatic antiresorptive therapy was generally well tolerated and was associated with clinically manageable disease courses despite heterogeneous biochemical trajectories, although five patients required individualized treatment with both agents (denosumab and zoledronate). Switches or additions were performed to support metabolic stabilization, particularly with zoledronate, or to escalate treatment to denosumab in cases of insufficient pain control especially in cases of persistent biochemical activity. However, within the treated subgroup one MAS patient with severe skeletal involvement experienced a clinically significant rebound period during treatment.

**Fig. 4:**
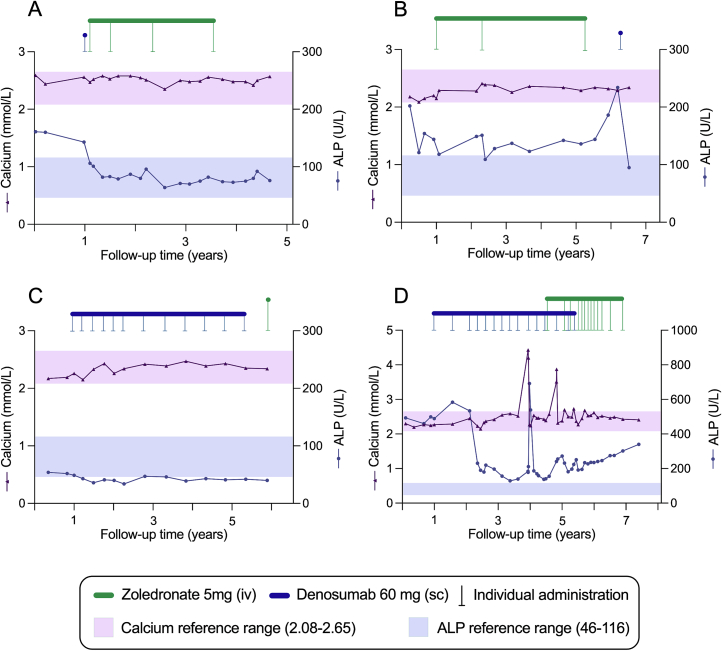
Sequential antiresorptive therapy and rebound risk in fibrous dysplasia/McCune-Albright syndrome. (A–D) Time course of plasma calcium and alkaline phosphatase (ALP) during individualized antiresorptive therapy. (A–C) Patient no. 6, 8, and 5 illustrate largely clinically stable treatment courses despite heterogeneous biochemical trajectories. (D) Patient no. 1 experienced severe hypercalcemia after delayed denosumab administration, consistent with a rebound phenomenon. Follow-up time is shown relative to the individual observation period of each patient.

The individualized treatment courses illustrate heterogeneous long-term metabolic trajectories over 6 ± 1 years, including three representative largely clinically stable treatment courses and one high-risk rebound course (Fig. 4), with additional longitudinal OC and DPD/Cr trajectories shown in Supplementary Fig. 4. Patient no. 6 demonstrated a marked reduction in ALP and OC following denosumab initiation. After transition to zoledronate therapy, calcium metabolism and most bone turnover parameters remained largely stable, although OC increased again towards the end of the zoledronate treatment interval without corresponding changes in calcium or DPD/Cr (Fig. 4A; Suppl. Fig. 4). In patient no. 8, calcium metabolism remained stable throughout follow-up despite persistently elevated ALP values during long-term zoledronate therapy, whereas OC values remained largely within the reference range. After later biochemical reactivation of bone turnover, denosumab escalation again reduced ALP levels without clinically relevant hypercalcemia during the observed follow-up period (Fig. 4B; Suppl. Fig. 4). Patient no. 5 exhibited stable low bone turnover activity and calcium levels throughout follow-up, while therapy was primarily continued because of symptomatic benefit and pain control rather than markedly elevated turnover markers (Fig. 4C).

In contrast to these clinically stable treatment courses, one young MAS patient with high pre-therapy ALP (489 U/L) experienced repeated reactivations of bone turnover and a severe rebound episode with hypercalcemia (4.43 mmol/L) and acute renal failure after a delay of approximately five weeks beyond the scheduled denosumab administration interval. Prior to this event, the patient had received denosumab 60 mg every three months. Following the rebound episode, clinical stabilization was achieved and biochemical control improved through acute denosumab administration followed by intensified sequential denosumab and zoledronate therapy (cumulative doses: denosumab 360 mg over 17 months; zoledronate 55 mg over 21 months) (Fig. 4D).

Together, these treatment courses demonstrate heterogeneous but largely clinically manageable long-term biochemical trajectories under individualized antiresorptive therapy in FD/MAS. However, even short delays in denosumab dosing may precipitate clinically relevant rebound phenomena in high-burden MAS.

## 4 Discussion

In this single-center cohort study of patients with FD/MAS, we observed that individualized antiresorptive therapy with denosumab and zoledronate appeared to be associated with largely clinically manageable disease courses despite heterogeneous biochemical trajectories and one clinically significant rebound case during follow-up. Moreover, almost 80% of treated individuals who completed a baseline pain assessment reported decreased pain during the course of treatment. Taken together, our findings suggest that antiresorptive therapy does not merely modulate surrogates of bone turnover but may also be associated with symptomatic benefit in FD/MAS, provided that treatment is tailored to disease burden and individual response.

To contextualize these findings, FD/MAS results from postzygotic activating mutations in the GNAS gene, leading to impaired osteoblast differentiation and increased osteoclast activity (Boyce and Collins, 2020; Schwindinger et al., 1992; Weinstein et al., 1991; Marie et al., 1997). Together, these alterations produce the characteristic fibro-osseous lesions composed of structurally unstable woven bone embedded in fibrous tissue (Raimondo et al., 2020; Marie, 2001). Elevated bone turnover markers reflect these underlying pathophysiological changes and are a hallmark of the disease. However, a direct link between bone turnover and pain remains uncertain, as several studies, including ours, have failed to show consistent correlations (Meier et al., 2023; Florenzano et al., 2019; Golden et al., 2024). This underlines the multifactorial nature of FD-related pain, which may involve mechanical stress, deformities, microfractures, or neuropathic mechanisms (Majoor et al., 2019b).

While suppression of bone turnover is not a therapeutic goal in itself, changes in bone turnover markers can serve as useful indicators of disease activity and treatment response (Javaid et al., 2019; van der Bruggen et al., 2021). In this context, several case series and small studies have suggested that antiresorptive drugs such as denosumab and zoledronate can reduce pain in FD/MAS patients, albeit with variable efficacy (Bertin et al., 2023; Trojani et al., 2023; Majoor et al., 2019a; Valadares et al., 2022). Within our study population, both drugs were used either sequentially or individually, depending on clinical need. Pain improvement was reported by almost 80% of treated patients. Proposed mechanisms of analgesic benefit under antiresorptive therapy include modulation of osteoclast activity, microdamage accumulation, and bone remodeling dynamics (Tucker-Bartley et al., 2023; Chapurlat et al., 2012). However, the exact mechanisms remain incompletely understood, as no significant longitudinal reduction in DPD/Cr was observed in our cohort to support reduced bone resorption as the main explanation for the analgesic effects.

Among bone turnover markers, ALP is of particular interest, as serum ALP correlates with skeletal burden and disease severity (Collins et al., 2005; Wang et al., 2019; Majoor et al., 2017). Similarly, we found significant but moderate correlations between lesion number and ALP, bALP, as well as OC in our cohort, whereas DPD/Cr did not correlate with lesion number. Consistent with previous studies (Bertin et al., 2023; Majoor et al., 2019a; Valadares et al., 2022; Ganda and Seibel, 2014), antiresorptive therapy led to significant suppression of bone turnover, particularly for ALP, bALP, and OC. The mean ALP reduction of approximately 44% was comparable to prior reports showing reductions of 20–40% with zoledronate and up to 63% with denosumab (Majoor et al., 2019a; Valadares et al., 2022; Ganda and Seibel, 2014; Chapurlat and Legrand, 2021), though direct comparisons are limited by small sample sizes and heterogeneous study designs. In contrast, DPD/Cr did not decrease significantly, possibly reflecting its weaker association with disease burden, greater longitudinal variability, and the limited sample size of the treated subgroup. Similar findings have previously been reported in FD/MAS, where bone formation markers appeared to show more consistent associations with lesion activity and treatment response than resorption markers (Bertin et al., 2023). This may reflect the predominantly fibro-osseous and bone formation driven pathology of FD/MAS, in which formation markers may more closely reflect lesion activity (Boyce and Collins, 2020). Besides treatment efficacy, long-term safety is of major importance. In this analysis, no cases of osteonecrosis of the jaw, hypocalcemia, atypical femoral fractures, or recurrent infections were observed, and only mild transient side effects occurred, mainly after zoledronate administration. Overall, most treated patients showed a clinically stable and manageable treatment course based on pain assessment and bone turnover markers, although some required drug switches to achieve improved pain control or adequate biochemical response, consistent with an individualized treatment approach in FD/MAS. However, our data also highlight a clinically relevant rebound risk associated with denosumab, particularly in a young adult MAS patient with high baseline bone turnover. Similar cases have been reported in the literature, usually in young MAS patients with a high disease burden (Meier et al., 2021; Boyce et al., 2012; de Castro et al., 2023). The rebound phenomenon observed after denosumab discontinuation likely reflects rapid reactivation of osteoclast activity following renewed RANKL signaling after withdrawal of RANKL inhibition, resulting in excessive bone resorption (Ferrari and Langdahl, 2023; Sølling et al., 2023). In FD/MAS, this process could be amplified by the disease-specific microenvironment. Constitutive Gsα activation together with increased local RANKL and IL-6 expression may create conditions permissive for exaggerated osteoclastic reactivation once denosumab effects decline (Yamamoto et al., 1996; de Castro et al., 2019; Riminucci et al., 2003). This mechanism may contribute to rebound hypercalcemia in our MAS patient, although the underlying pathophysiology remains incompletely understood. Importantly, prior bisphosphonate exposure has been suggested to reduce rebound risk (Uebelhart et al., 2017). However, our patient had received long-term pamidronate therapy before denosumab yet still developed a severe rebound. This implies that protective effects of bisphosphonates may not generally apply to FD/MAS, particularly not in patients with very high baseline bone turnover.

We acknowledge several limitations to our study. First, the retrospective observational design allows only associations to be identified, not causal relationships. Thus, prospective, multicenter studies with standardized antiresorptive regimens and predefined biochemical and patient-reported endpoints are required. Furthermore, treatment was not standardized: although all patients received either 60 mg denosumab or 5 mg zoledronate, dosing intervals and therapy switches varied according to clinical and biochemical response. This heterogeneity, however, reflects individualized treatment strategies for rare bone disorders like FD/MAS, characterized by diverse subtypes and variable courses (Javaid et al., 2019). In addition, characterization of rebound phenomena was limited by the retrospective design, heterogeneous monitoring intervals, and the small number of clinically significant rebound events. Consequently, biochemical reactivations and rebound-associated complications could only be assessed descriptively rather than through predefined or systematically quantified criteria. Nevertheless, the longitudinal real-world follow-up provides clinically relevant insights into individualized rebound-associated treatment trajectories in a rare disease setting for which systematic long-term data remain scarce. Finally, the dataset itself was heterogeneous, with incomplete data available for some patients, including quantitative pain assessments, which limited more detailed longitudinal analyses of pain response under therapy. Consequently, pain-related outcomes could not be assessed in a fully standardized manner across the entire treated cohort. Nevertheless, this study provides valuable insights into a very rare disease for which therapeutic evidence is extremely limited, and to our knowledge, it is the first to report on the use of both denosumab and zoledronate within the same patient cohort. To address the limitations of small and heterogeneous cohorts, international disease registries and standardized datasets have been developed as useful tools to harmonize data collection and support natural history research in FD/MAS (Priego Zurita et al., 2025).

In conclusion, FD/MAS is a rare and still incompletely understood disorder with highly heterogeneous clinical manifestations. Our data suggest that individualized therapy with denosumab and zoledronate may contribute to stabilization of bone turnover and symptomatic improvement in selected patients. However, denosumab treatment may be associated with clinically relevant rebound phenomena, particularly in young MAS patients with extensive skeletal burden and high bone turnover. Careful long-term monitoring and strict adherence to dosing intervals therefore appear particularly important in FD/MAS patients receiving denosumab. In contrast, zoledronate may represent an option for long-term stabilization or consolidation therapy in selected patients. Given the rarity of FD/MAS and the lack of high-quality evidence, prospective multicenter studies or randomized controlled trials are needed to establish safe and effective treatment strategies.

## CRediT authorship contribution statement

**Tonio Lipkow:** Writing – review & editing, Writing – original draft, Visualization, Methodology, Investigation, Formal analysis, Data curation. **Mikolaj Bartosik:** Writing – review & editing, Visualization. **Johann Sprick-Schütte:** Writing – review & editing, Formal analysis. **Florian Barvencik:** Writing – review & editing. **Michael Amling:** Writing – review & editing. **Ralf Oheim:** Writing – review & editing, Writing – original draft, Supervision, Project administration, Investigation, Data curation, Conceptualization.

## Ethics statement

The authors of this manuscript confirm that they adhere to the ethical guidelines for authorship and publication in *Bone Reports*. This retrospective study was conducted in accordance with local ethical guidelines and the Declaration of Helsinki. Written informed consent was obtained from all patients for the retrospective analysis of data derived from routine clinical measurements performed during outpatient clinic visits.

## Funding

This study received funding from the DFG (10.13039/501100001659German Research Foundation) within the Clinical Research Unit 5029 to MA, FB, and RO (project number 517063424).

## Declaration of competing interest

The authors declare the following financial interests/personal relationships which may be considered as potential competing interests: Ralf Oheim, Florian Barvencik and Michael Amling report financial support was provided by 10.13039/501100001659German Research Foundation. Ralf Oheim reports a relationship with Kyowa Kirin that includes: board membership, funding grants, and speaking and lecture fees. Ralf Oheim reports a relationship with UCB that includes: board membership, funding grants, and speaking and lecture fees. Ralf Oheim reports a relationship with Inozyme Pharma that includes: board membership, funding grants, and speaking and lecture fees. Ralf Oheim reports a relationship with Ipsen that includes: board membership and speaking and lecture fees. Ralf Oheim reports a relationship with Pharmacosmos that includes: board membership and speaking and lecture fees. Ralf Oheim reports a relationship with Mereo that includes: board membership and speaking and lecture fees. Florian Barvencik reports a relationship with Alexion that includes: funding grants and speaking and lecture fees. Florian Barvencik reports a relationship with UCB that includes: funding grants and speaking and lecture fees. Florian Barvencik reports a relationship with Diasorin that includes: speaking and lecture fees. Mikolaj Bartosik reports a relationship with Fresenius that includes: travel reimbursement. If there are other authors, they declare that they have no known competing financial interests or personal relationships that could have appeared to influence the work reported in this paper.

## Data Availability

The data underlying this article will be shared upon reasonable request to the corresponding author. However, some data are restricted due to data privacy policies.
