# Supplementary material for: Individualized antiresorptive therapy in fibrous dysplasia and McCune-Albright syndrome: A retrospective cohort study
Source: Bone Rep. 2026 Jun 19;30:101933. doi: 10.1016/j.bonr.2026.101933 (PMC13314581; doi:10.1016/j.bonr.2026.101933)
Supplement: Supplementary file 1 — Supplementary material [file mmc1.pdf]

Supplementary information

Article title:
Individualized Antiresorptive Therapy in Fibrous Dysplasia and McCune-Albright Syndrome: A Retrospective Cohort Study

Journal name:
Bone Reports

Author names:
Tonio Lipkow[1], Mikolaj Bartosik MD[1], Johann Sprick-Schütte[1], Florian Barvencik MD[1], Michael Amling MD[1], and Ralf Oheim MD[1]

Corresponding Author:
Ralf Oheim, MD, Department of Osteology and Biomechanics, University Medical Center Hamburg-Eppendorf, Martinistrasse 52, 20246 Hamburg, Germany, Email: r.oheim@uke.de

Supplementary Table 1 Additional bone turnover markers. Abbreviations: monostotic fibrous dysplasia (MFD), polyostotic fibrous dysplasia (PFD), McCune–Albright syndrome (MAS), Mazabraud syndrome (MS), parathyroid hormone (PTH), 25-hydroxyvitamin D (25(OH)D), procollagen type 1 N-terminal propeptide (P1NP), and serum collagen type I C-terminal telopeptide (CTX). Statistically significant differences are shown with exact p-values in bold.

* Group comparisons were performed for MFD, PFD, and MAS; MS was excluded due to small sample size.

Additional bone turnover markers

	MFD (n = 21)	PFD (n = 10)	MAS (n = 9)	MS (n = 2)	P-value*
Bone turnover markers, mean (SD)					
PTH (ng/L)	53.45 (29.78)	43.94 (20.53)	67.95 (38.01)	34.51 (17.61)	0.245
25(OH)D (ng/l)	33.76 (17.90)	27.65 (8.48)	31.88 (14.97)	30.45 (13.51)	0.588
P1NP (µg/L)	67.16 (26.40)	58.13 (11.34)	286.5 (65.54)	51.30 (0)	**0.015**
CTX (µg/L)	0.33 (0.18)	0.24 (0.06)	0.67 (0.33)	0.15 (0)	0.181

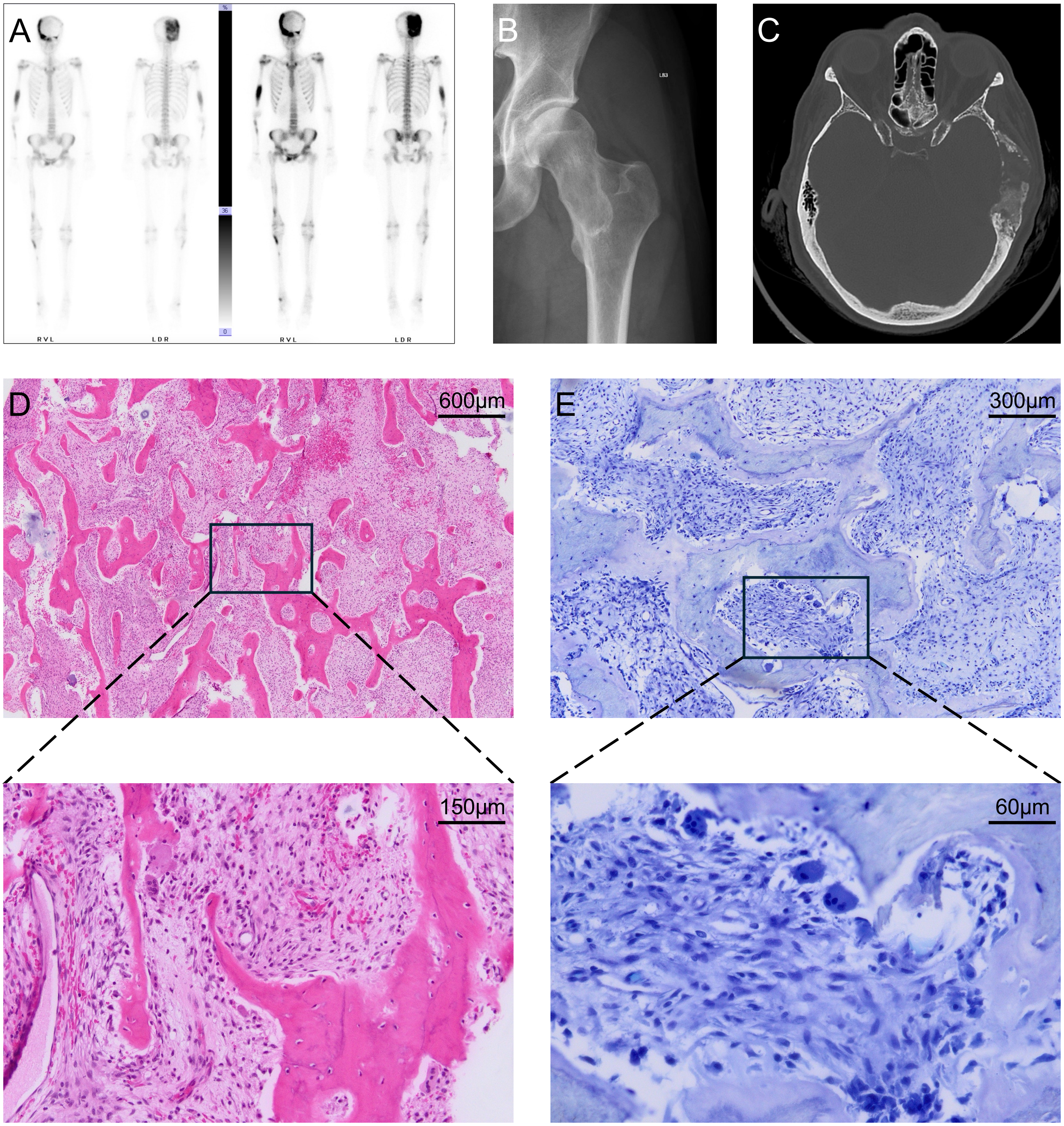

Supplementary Fig. 1 Representative radiological and histological findings of fibrous dysplasia/McCune-Albright syndrome (FD/MAS). (A) Whole-body skeletal scintigraphy of a patient with MAS, demonstrating multiple FD lesions in the cranium, humerus, femur, pubic bone, and tibia. (B) X-ray image of an FD lesion in the proximal femur with the typical “ground-glass” appearance. (C) Computed tomography (CT) scan of an FD lesion in the temporal bone. (D) Histological image from a femoral bone biopsy of a MAS patient, showing irregular, branching trabeculae with the characteristic “Chinese letter” pattern and spindle-shaped fibrous stroma (hematoxylin and eosin staining). (E) Toluidine blue staining demonstrating increased osteoclast numbers and elevated bone remodeling activity.

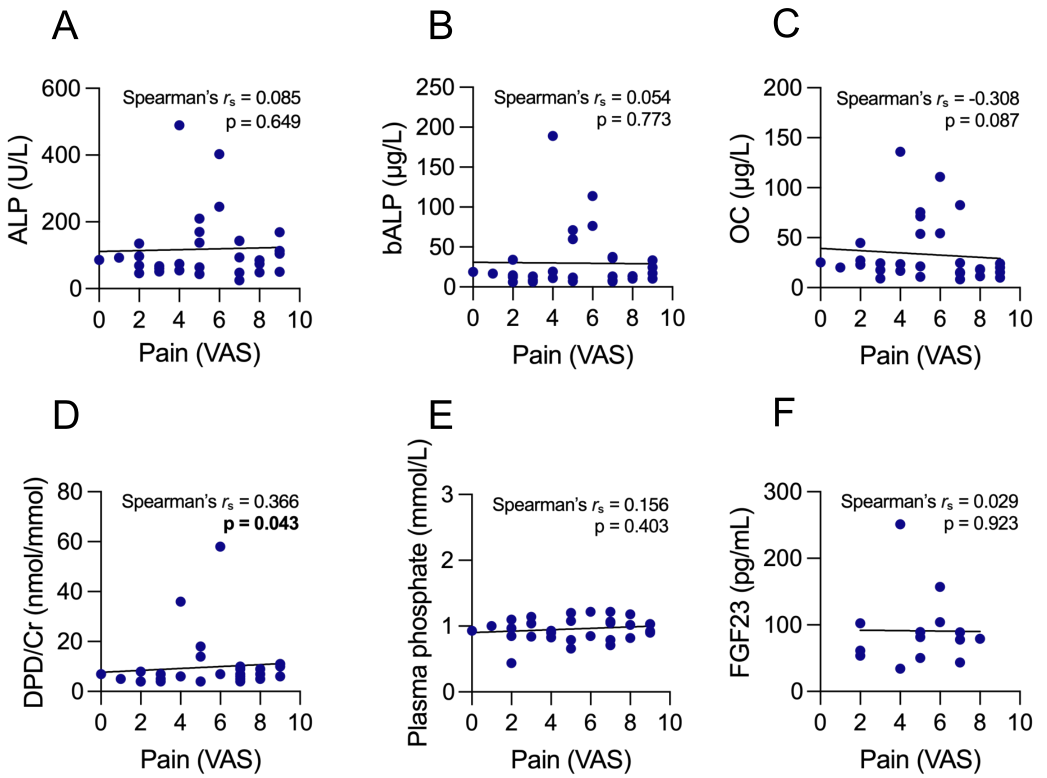

Supplementary Fig. 2 Associations between pain and baseline bone turnover. (A-D) Correlations between visual analogue scale (VAS) pain scores and baseline bone turnover markers, including alkaline phosphatase (ALP), bone-specific alkaline phosphatase (bALP), osteocalcin (OC), and urinary deoxypyridinoline/creatinine (DPD/Cr). (E-F) Additional correlations with parameters of phosphate metabolism: plasma phosphate and intact fibroblast growth factor 23 (FGF23). Statistically significant correlations are shown with exact p-values in bold.

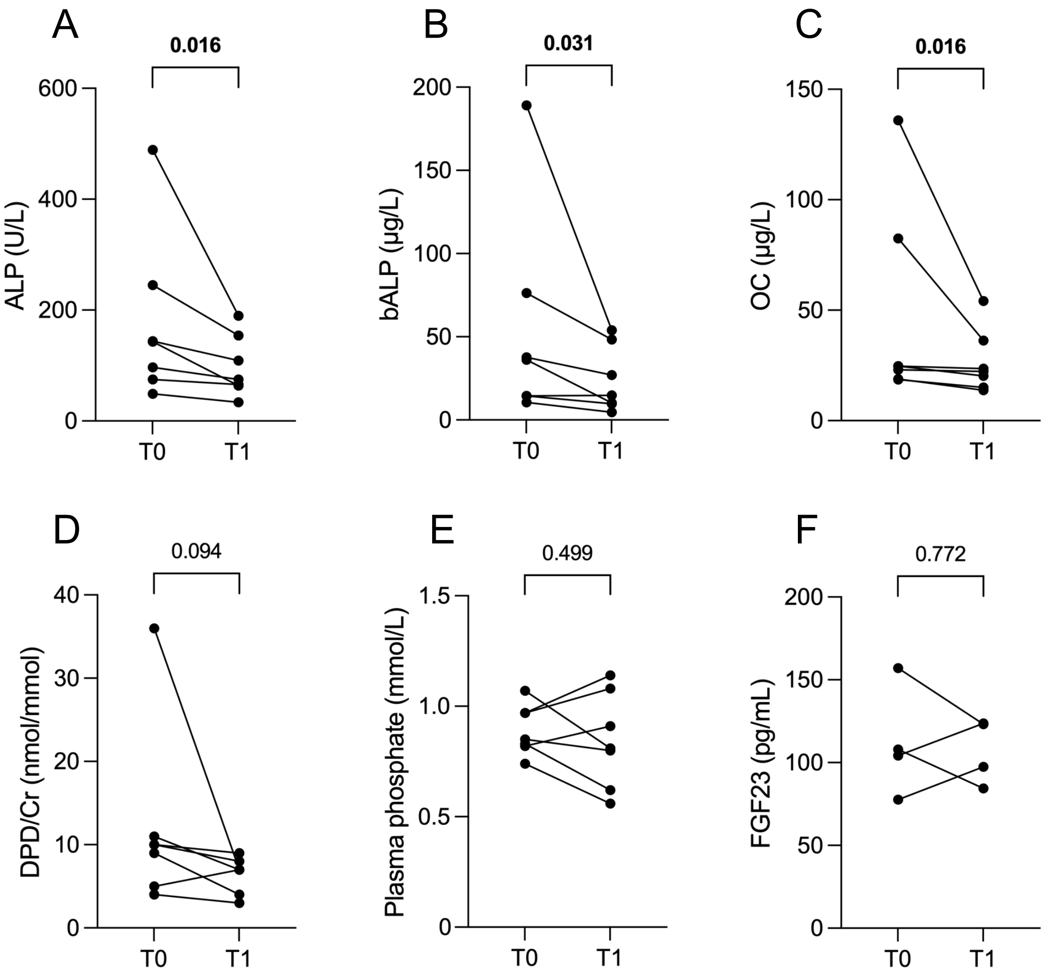

Supplementary Fig. 3 Individual longitudinal responses of bone metabolism markers to antiresorptive therapy. (A-D) Individual paired baseline (T0) and follow-up (T1; mean follow-up 16 months) values for alkaline phosphatase (ALP), bone-specific alkaline phosphatase (bALP), osteocalcin (OC), and urinary deoxypyridinoline/creatinine (DPD/Cr). (E-F) Individual paired values for plasma phosphate and intact fibroblast growth factor 23 (FGF23). Lines connect measurements from the same patient. This figure complements the group-level analyses shown in Figure 3. Statistically significant differences are shown with exact p-values in bold.

Supplementary Fig. 4

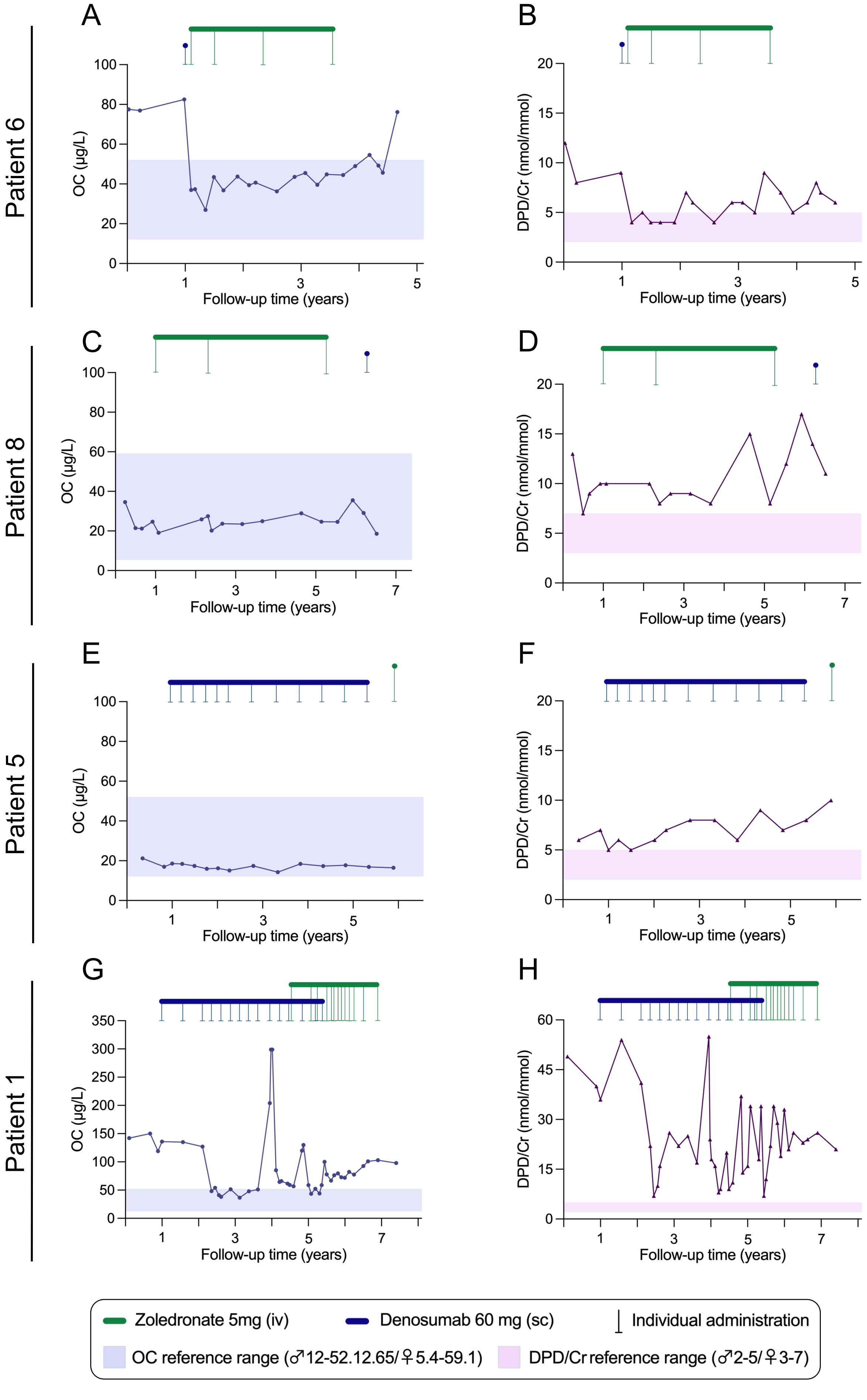

Supplementary Fig. 4 Additional longitudinal bone turnover marker trajectories in sequentially treated patients with fibrous dysplasia/McCune-Albright syndrome. (A-H) Time course of osteocalcin (OC) and urinary deoxypyridinoline/creatinine (DPD/Cr) during individualized antiresorptive therapy. Male (♂) and female (♀) reference ranges are indicated separately in the figure legend. Follow-up time is shown relative to the individual observation period of each patient.
